# Criteria for bioreactor comparison and operation standardisation during process development for mammalian cell culture

**DOI:** 10.1186/1753-6561-5-S8-P47

**Published:** 2011-11-22

**Authors:** Oscar Platas Barradas, Uwe Jandt, Linh Da Minh Phan, Mario Villanueva, Alexander Rath, Udo Reichl, Eva Schräder, Sebastian Scholz, Thomas Noll, Volker Sandig, Ralf Pörtner, An-Ping Zeng

**Affiliations:** 1 Institute of Bioprocess and Biosystems Engineering, Hamburg University of Technology, Hamburg, D-21073, Germany; 2 Bioprocess Engineering, Max Planck Institute for Dynamics of Complex Technical Systems, Magdeburg, D-39106, Germany; 3 Faculty of Technology, AG Zellkulturtechnik, Bielefeld University, Bielefeld, D-33501, Germany; 4 ProBioGen AG, Berlin, D-13086, Germany

## Background

Development of bioprocesses for animal cells has to deal with different bioreactor types and scales. Bioreactors might be intended for generation of cell inoculum and production, research, process development, validation or transfer purposes. During these activities, not only the difficulty of up- and downscaling might lead to failure of consistency in cell growth, but also the use of different bioreactor geometries and operation conditions. In such cases, the criteria for bioreactor design and process transfer should be carefully evaluated in order to avoid an erroneous transfer of cultivation parameters.

In this work, power input, mixing time, impeller tip speed, and Reynolds number have been compared systematically for the cultivation of the human cell line AGE1.HN^®^ within three partner laboratories using five different bioreactor systems. A common process window for mixing time in the range of 8 – 13 s has been found in bioreactors having significant differences in their inner geometries. The obtained results are employed for process standardisation and transfer between research institutions.

## Cell culture in laboratory bioreactors with different inner geometries

Finding conditions for consistent cultivation of mammalian cells in bioreactors is not an easy task. For standard stirred tanks, correlations existing in literature can be used in order to predict operation conditions for process transfer purposes. However, if the inner geometry of two bioreactors cannot be compared within tolerance ranges, the characterization of the bioreactor hydrodynamics becomes necessary.

For this work, five geometrically different bioreactors were used, which are operated within three partner laboratories for data generation during research on Systems Biology. Characterization of the bioreactor hydrodynamics was performed with the main goal of finding a relationship between process transfer criteria and cell growth in the systems.

## Bioreactor characterization

Following criteria were considered for characterization of bioreactor hydrodynamics.

**(1) Power input (*P*/*V*):** Power numbers *Np* were calculated from *Np* = *f*(*Re*) correlations available in literature [1-3]. Corrections for *Np* were considered due to geometry deviations from a *standard* configuration [4-7]. Volumetric power inputs were calculated according to Equation 1:(1)

**(2) Mixing time (*Θ_94.5_*):** This criterion was obtained from the decolourization of a *I/KI* solution of after addition of *Na_2_S_2_O_3_*. Starch was added previously to the bioreactor. Decolourization time course was video recorded. The resulting videos were computer analyzed, and *Θ_94.5_* was obtained after gray-scale conversion and measurement of loss of saturation (MATLAB, MathWorks).

**(3) Impeller tip speed (*u_tip_*):** calculated according to Equation 2.(2)

**(4) Reynolds Number at impeller tip (*Re_i_*):** calculated according to equation 3:(3)

The specific growth rate ***μ_max_*** was employed as indicator for comparison of bioreactor performance. The use of *μ_max_* made the comparison of the two cell line clones possible, despite the differences in initial cell densities during bioreactor culture.

## Relationship between cell growth and process transfer criteria

**Figure 1:**
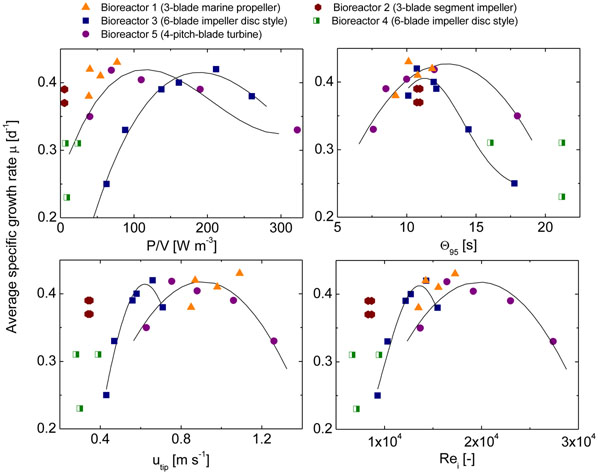
**Relationship between maximum specific growth rate *μ_max_* and values for process transfer during bioreactor culture**: a) Power input, b) Mixing time, c) Impeller tip speed, and d) Reynolds number. Curve fitting for bioreactors 3 (Black square) and 5 (Purple circle).

Figure 1 shows the dependency of the *μ_max_* on process transfer criteria. A process window for mixing time values between 8 and 13 seconds can be identified as common for all bioreactors, where the smallest deviation in *µ_max_* between different bioreactors can be observed.

## Conclusions

Criteria for process transfer were analyzed during the cultivation the human production cell line AGE1.HN. Growth was compared within ranges for power input, mixing time, impeller tip speed and Reynolds number. Maximum specific growth rates were observed for AGE1.HN cells at a common mixing time range of 8 - 13 seconds for all cultivation systems. This criterion was observed to be a reference for consistency of results within laboratory bioreactors with different internal geometry.

Funding by the BMBF, Grand Nr. 0315275A is gratefully acknowledged.

## Nomenclature

*d_i_*    impeller diameter    [m]

*M*    degree of mixing

*N*    agitation speed    [rpm]

*P* = *NpρN^3^d_i_^5^*    power input    [W m^-3^]

*Re_i_*    Reynolds number at impeller tip    [-]

*u_tip_*    impeller tip speed    [m s^-1^]

*V*    working volume    [m^3^]

## Greek letters

*Θ*    mixing time    [s]

*μ_max_*    specific growth rate    [d^-1^]

*ρ*    density    [kg m^-3^]

*η*    dynamic viscosity    [kg m^-1^ s^-1^]
